# Impact of HIV Pre‐Exposure Prophylaxis on HIV Incidence and Hepatitis B Outcomes Among Sex Workers Living With Hepatitis B: A Modelling Analysis in Côte D'Ivoire

**DOI:** 10.1002/jia2.70217

**Published:** 2026-10-26

**Authors:** Amir M. Mohareb, Arthur Y. Kim, Menan Gérard Kouamé, Marcellin Nouaman, Patrick A. Coffie, Maya Hajny Fernandez, Virginia R. Talbot, Ruitian Xeno Hu, Anne M. Neilan, Anders Boyd, Kenneth A. Freedberg, Joseph Larmarange, Emily P. Hyle

**Affiliations:** ^1^ Medical Practice Evaluation Center Massachusetts General Hospital Boston Massachusetts USA; ^2^ Division of Infectious Diseases Massachusetts General Hospital Boston Massachusetts USA; ^3^ Department of Medicine Harvard Medical School Boston Massachusetts USA; ^4^ Programme PAC‐CI Abidjan Côte d'Ivoire; ^5^ Département de Médecine et Spécialités Médicales, UFR des Sciences Médicales Université Félix Houphouët‐Boigny Abidjan Côte d'Ivoire; ^6^ Department of Pediatrics Harvard Medical School Boston Massachusetts USA; ^7^ Amsterdam Institute for Immunology and Infectious Diseases Amsterdam Netherlands; ^8^ Department of Infectious Diseases Public Health Service of Amsterdam Amsterdam Netherlands; ^9^ Department of Infectious Diseases Bern University Hospital University of Bern Bern Switzerland; ^10^ Division of General Internal Medicine Massachusetts General Hospital Boston Massachusetts USA; ^11^ Centre Population et Développement Université Paris Cité Université Sorbonne Paris Nord, IRD, Inserm Paris France

**Keywords:** Africa, hepatitis B virus, hepatitis, HIV, pre‐exposure prophylaxis (PrEP), sex workers

## Abstract

**Introduction:**

Nearly 10% of sex workers in sub‐Saharan Africa live with chronic hepatitis B virus (HBV). HIV pre‐exposure prophylaxis (PrEP) using tenofovir/emtricitabine (TDF/FTC) has not been studied in this population, possibly due to concerns of hepatitis flares following TDF/FTC cessation.

**Methods:**

Using a microsimulation model of HIV and HBV disease and data from 2016 to 2024, we compared the clinical impact of *TDF/FTC* as PrEP versus *no TDF/FTC* among women who sell sex with HBV in Côte d'Ivoire. We incorporated the anti‐HBV effects of TDF/FTC and the increased probability of hepatitis flares and HBV surface antigen (HBsAg) loss following TDF/FTC cessation. We estimated input parameters from published literature: HIV incidence (1.9–3.0 per 100 person‐years), PrEP adherence (55%), disengagement from PrEP care (4% per month for the first 12 months, then 12.5% per month), hepatitis flare incidence (2.0−4.0 per 100 person‐years) and rate of HBsAg loss (1.0 per 100 person‐years), which both increase following TDF/FTC cessation. Model outcomes included cumulative HIV incidence, average hepatitis flares, HBsAg loss and survival over 2, 5 and 10 years. We conducted sensitivity analyses varying HIV incidence, PrEP adherence, disengagement from PrEP care and hepatitis flare rate.

**Results:**

Compared with *no TDF/FTC*, *TDF/FTC* would reduce cumulative HIV incidence: 4.1% versus 5.6% at 2 years and 10.1% versus 12.7% at 5 years. *TDF/FTC* would result in more hepatitis flares (mean 0.76 vs. 0.19 per person) and more HBsAg loss (8.4% vs. 5.7%) than *no TDF/FTC* after 5 years. Two‐ and five‐year survival would be similar between the two strategies, and *TDF/FTC* would improve survival over longer horizons. Improving adherence and reducing disengagement from care would increase clinical benefits. Even with high disengagement from care and high risk of hepatitis flares, *TDF/FTC* would reduce HIV acquisition and improve overall long‐term survival.

**Conclusions:**

Women who sell sex with HBV in Côte d'Ivoire would benefit from TDF/FTC as PrEP to avoid HIV acquisition. The increased risk of hepatitis flares, while challenging to manage in resource‐limited settings, would be outweighed by the benefits of HIV prevention. Efforts should be made to scale up PrEP in sex workers, including women with chronic HBV.

## 1 Introduction

In sub‐Saharan Africa, the prevalence of chronic hepatitis B virus (HBV) can be as high as 8%–12% [1]. People with HBV who acquire HIV can experience accelerated liver disease, hepatocellular carcinoma (HCC) and early mortality, even when treated with antiretroviral therapy (ART) [2, 3, 4]. Therefore, prevention of HIV‐HBV coinfection is a public health priority [5]. Priority populations in sub‐Saharan Africa, such as the estimated 2.5 million sex workers across the continent, incur a high risk of HIV‐HBV coinfection [6, 7, 8]. Sex workers living with HBV could benefit from HIV pre‐exposure prophylaxis (PrEP) to reduce the risk of HIV acquisition [5, 9, 10]. However, tenofovir cessation can prompt HBV reactivation [9, 11, 12], which has an associated risk of acute hepatitis (i.e. hepatitis flares) and can lead to hospitalization, acute liver failure, and death [13, 14]. In some cases, HBV reactivation in the context of tenofovir cessation can also result in HBV surface antigen (HBsAg) loss, which is associated with lower rates of liver‐related morbidity and mortality [15]. Nearly all clinical trials of oral PrEP have excluded people living with HBV [11], so it is not known whether the HBV‐specific risks of TDF/FTC discontinuation outweigh its benefits for HIV prevention [16].

Like many countries in sub‐Saharan Africa, Côte d'Ivoire is endemic for HBV and has a high prevalence (5.6%) of HIV among the estimated 30,000 sex workers in the country [17]. Women who sell sex often use PrEP in seasonal cycles, corresponding to periods where they are at increased risk of HIV acquisition [18]. For people with chronic HBV, the increased risk of hepatitis flares following TDF/FTC cessation may dissuade clinicians from prescribing oral PrEP despite its established benefits in preventing HIV. Using a computer microsimulation model, our objective was to examine the trade‐offs between prevention of HIV acquisition and increased risk of HBV flares when TDF/FTC is used as PrEP among women who sell sex with HBV in Côte d'Ivoire.

## 2 Methods

### 2.1 Analytic Overview

We used the Cost Effectiveness of Preventing AIDS Complications‐International (CEPAC‐I) model, a simulation model of HIV [19, 20, 21], to project the clinical impact of oral PrEP among sex workers in Côte d'Ivoire. We developed a novel chronic HBV disease module to simulate the impact of TDF/FTC on HBV‐related outcomes, including hepatitis flares, as well as incident HIV [22]. We simulated a cohort of adult women living with chronic HBV who sell sex and are at risk of HIV acquisition (i.e. all have HBV, and none have HIV at model start). We tested two strategies of care: (1) *TDF/FTC* as oral PrEP; (2) *no TDF/FTC*. We populated the model with country‐specific HIV and PrEP parameters from Côte d'Ivoire, including data derived from the PRINCESSE study, a single‐arm pragmatic clinical trial evaluating the feasibility of a comprehensive sexual health package for sex workers in Côte d'Ivoire (2019–2024) [23]. Other data for the model are related to the effects of PrEP on HIV acquisition and HBV outcomes, mostly sourced between 2016 and 2024. We projected HIV incidence, hepatitis flares, HBsAg loss and survival. We evaluated uncertainty in model results through one‐way and multi‐way sensitivity analyses, following guidance from the International Society for Pharmacoeconomics and Outcomes Research and the Society for Medical Decision Making (ISPOR‐SMDM) Modeling Good Research Practices Task Force Working Group–6 [24]. This study was approved by the Mass General Brigham Human Research Committee, and no consent was required because no data were collected from human subjects.

### 2.2 HBV Model Structure

We developed natural history states for chronic HBV within the CEPAC‐I simulation model (Figure S1): (1) no fibrosis; (2) fibrosis; (3) cirrhosis; (4) HCC; (5) end‐stage liver disease (ESLD); (6) HBsAg loss; (7) hepatitis flare; and (8) death [22]. At model entry, simulated people draw for the health states associated with chronic HBV and face monthly transition probabilities of developing HBV‐related complications. HBV‐active antiviral therapy with TDF/FTC reduces the probability of HBV disease progression [25]. People who lose HBsAg are considered cured of chronic HBV [26]. Individuals with HBV have a risk of temporarily transitioning to the hepatitis flare state, which is characterized by a higher risk of death. People who have taken TDF/FTC for at least 4 consecutive months incur an increased probability of developing a hepatitis flare if they stop TDF/FTC. This risk peaks in the month after cessation and then diminishes exponentially over 72 months until it reaches the baseline off‐treatment risk [15, 27, 28, 29]. Each simulation month, people have a probability of death from HBV‐related or non‐HBV‐related causes.

### 2.3 HIV Model Structure

Sex workers with HBV enter the model without HIV and face monthly risks of HIV acquisition. Individuals acquiring HIV draw for an initial CD4 count and subsequently experience HIV disease progression, including CD4‐stratified risks of opportunistic infections. Treatment with ART reduces the risk of HIV disease progression and opportunistic infections. We defined PrEP effectiveness as the risk reduction in HIV acquisition, which is represented as a cross‐product of PrEP efficacy (i.e. HIV risk reduction under ideal pharmacologic conditions) and PrEP adherence (i.e. percentage of days with bioavailable TDF/FTC for HIV prevention) [21].

### 2.4 Cohort Characteristics

We parameterized the model with cohort characteristics of sex workers in Côte d'Ivoire, including the PRINCESSE study [23]. All simulated persons are women, and the mean age at model start is 21 years (standard deviation [SD], 1 year) (Table 1) [30]. We initiated the model with the following HBV health state distribution: 94% no fibrosis, 5% moderate or severe fibrosis, 1% cirrhosis, 0% HCC and 0% ESLD [31, 32]. The limited data evaluating all‐cause mortality in women who sell sex suggest a markedly increased risk of death compared with the age‐matched general population (Table S1) [33]. We, therefore, applied a relative mortality ratio of 8.0 to the background age‐stratified mortality risk for women in Côte d'Ivoire up to the age of 40 years in the base case scenario, and we explored the impact of lower rates of mortality in sensitivity analyses, described below. We used a simulation size of 8,000,000, which produced convergent results in outcome projections and thus indicated the results were not varying due to stochastic variation.

**TABLE 1:** Base case input parameters for an analysis of *TDF/FTC* versus *no TDF/FTC* as HIV pre‐exposure prophylaxis (PrEP) among women who sell sex with chronic HBV in Côte d'Ivoire.

Variable	Base case value		Reference
**Cohort characteristics**			
Age, year, mean (SD)	21 (1)		[34]
HBV health state, %			[31, 32]
No/mild fibrosis	94		
Moderate/severe fibrosis	5.5		
Cirrhosis	0.5		
HCC	0		
ESLD	0		
**HIV**			
HIV incidence, by age, per 100 person‐years			[34, 35]
< 25 years	3.0		
25−30 years	2.4		
31−35 years	1.9		
36−40 years	1.5		
41−55 years	0.8		
PrEP			
Efficacy	95%		[36]
Adherence	55%		[36]
Disengagement from PrEP care (monthly probability)			[37]
Within 12 months of use	4.0%		
At or after 12 months of use	12.5%		
Return to PrEP care (monthly probability)			
Within 6 months of cessation	1.0%		[18, 37]
6–12 months after cessation	3.0%		
>12 months after cessation	6.0%		
HIV testing frequency regardless of TDF/FTC	Every 5 months		[5, 23]
**HBV progression** (per 100 person‐years, at age 25)			
	Off TDF/FTC	Proportional risk reduction on TDF/FTC	
No fibrosis to fibrosis	1.50	0.37	[38]
Fibrosis to cirrhosis	6.56	0.37	[39, 40]
Cirrhosis to ESLD	6.00	0.59	[39, 40]
No fibrosis or fibrosis to HBsAg loss	1.00	1.00	[41]
Fibrosis to HCC	2.00	0.59	[42, 43]
HCC to ESLD	8.00	0.59	[40]
HBV‐specific mortality	Risk of death with HBV	Risk reduction on TDF/FTC	[40, 44]
Moderate/severe fibrosis	0.10	0.92	
Cirrhosis	6.02	0.92	
HCC	48.99	0.92	
ESLD	36.56	0.92	

Abbreviations: ESLD, end‐stage liver disease; HBsAg, hepatitis B virus surface antigen; HBV, hepatitis B virus; HCC, hepatocellular carcinoma; PrEP, pre‐exposure prophylaxis; SD, standard deviation; TDF/FTC, tenofovir disoproxil fumarate/emtricitabine.

### 2.5 HBV‐Related Inputs

We used published estimates of HBV disease progression from studies of HBV monoinfection: age‐stratified rates of incident fibrosis and cirrhosis (1.50−6.56 per 100 person‐years) and HCC (0.5−2.0 per 100 person‐years) [39, 45, 46]. People who take TDF/FTC for at least 12 months have a 63% risk reduction for fibrosis and cirrhosis and a 41% risk reduction for HCC and ESLD [25]. HBsAg loss occurs at 1.0 per 100 person‐years [47]. Incidence of hepatitis flares is 4.0 per 100 person‐years for people without cirrhosis and 7.9 per 100 person‐years for people with cirrhosis or ESLD [48]. People in the hepatitis flare state experience an initial eight‐fold increase in the likelihood of HBsAg loss [9, 48, 49, 50]. Continuous TDF/FTC use reduces the probability of hepatitis flares by 30% [48, 51].

### 2.6 HIV‐Related Inputs

We used data from the PRINCESSE study to estimate HIV incidence, PrEP adherence, disengagement in care and HIV screening. HIV incidence declines with older age: 3.0 per 100 person‐years until age 25 and decreasing to 0.8 per 100 person‐years for women 40 years or older (Table 1) [17, 34]. Oral PrEP is 95% effective at reducing HIV incidence and PrEP adherence at 55%, which combine for a 45% reduction in HIV incidence [21, 36]. People stop PrEP when disengaged from care at 4% per month during the first 12 months, subsequently increasing to 12.5% per month, as derived from the PRINCESSE study and other PrEP studies in sub‐Saharan Africa (Table S2 and Figure S2) [37]. To simulate cyclical PrEP use in sex workers, which often occurs based on seasonal employment and other risk factors [18, 37], we implemented a monthly probability of returning to PrEP care, which varies over time: 1.0% per month for the first 6 months after disengagement from care; 3.0% per month between 6 and 12 months; and 6.0% per month after 12 months (Figure S2) [18, 37, 52]. Simulated sex workers would complete HIV screening at 5‐month intervals, as derived from the PRINCESSE study [23].

### 2.7 Model Outcomes and Sensitivity Analysis

We projected 2‐ and 5‐year cumulative incidence of HIV, hepatitis flares, HBsAg loss, moderate or severe fibrosis, cirrhosis, HCC and ESLD. We additionally projected survival to 10 years. To examine the effect of parameter uncertainty on HIV and HBV outcomes, we performed one‐way sensitivity analyses on PrEP adherence, the probability of disengaging from care, the probability of returning to PrEP care, the probability of hepatitis flares, the risk of death associated with hepatitis flares and the incidence of HBsAg loss following TDF/FTC cessation (Table S3). When available, we used 95% confidence intervals from the published literature to guide choice of parameter ranges in the sensitivity analyses. For parameters with little published information on uncertainty, we adopted a conservative approach using a broad range in sensitivity analyses [24]. We conducted a two‐way sensitivity analysis on hepatitis flare probability and mortality associated with hepatitis flares. Compared with the base case scenario (hepatitis flare: ALT > two times the upper limit of normal), we assumed that more severe flares would be less frequent but associated with a higher risk of death. We also conducted a two‐way sensitivity analysis on hepatitis flare probability and likelihood of HBsAg loss following TDF/FTC cessation. Last, we examined scenarios with a higher starting age of cohort (mean age 35 years), lower HIV incidence rate (1.5 per 100 person‐years), lower background mortality (i.e. no relative mortality ratio applied) and 75% linkage to care following HIV diagnosis.

## 3 Results

### 3.1 HIV Incidence and Survival

*TDF/FTC* would result in lower cumulative HIV incidence compared with *no TDF/FTC* at 2 years (4.1% vs. 5.7%) and 5 years (10.1% vs. 12.7%) (Table 2). Survival differences would increase with time: at 10 years, the survival would be 73.8% with *TDF/FTC* versus 72.6% with *no TDF/FTC* (Table 2 and Figure S3).

**TABLE 2:** Two‐ and five‐year outcomes of *TDF/FTC* versus *no TDF/FTC* as PrEP in women who sell sex with chronic HBV in Cote d'Ivoire.

Strategy	HIV incidence (%)		Hepatitis flares (per person)		HBsAg loss (%)		Survival (%)		
	2 years	5 years	2 years	5 years	2 years	5 years	2 years	5 years	10 years
No TDF/FTC	5.7	12.7	0.08	0.19	2.3	5.7	96.2	89.4	72.6
TDF/FTC	4.1	10.1	0.28	0.76	2.9	8.4	96.2	89.4	73.8
Sensitivity analysis									
TDF/FTC with perfect retention in care	2.9	6.7	0.06	0.15	2.3	5.5	96.2	89.8	79.9

Abbreviations: HBV, hepatitis B virus; PrEP, pre‐exposure prophylaxis; TDF/FTC, tenofovir disoproxil fumarate/emtricitabine.

### 3.2 HBV‐Related Outcomes

There would be more hepatitis flares with *no TDF/FTC* compared with *TDF/FTC* for the first 6 months of the simulation. As people disengage from care and stop TDF/FTC, the number of hepatitis flares would rise in *TDF/FTC*, surpassing *no TDF/FTC* and peaking 20 months after PrEP start (Figure 1). The cumulative incidence of HBsAg loss would be higher in *TDF/FTC* compared with *no TDF/FTC*: 2.9% versus 2.3% after 2 years and 8.4% versus 5.7% after 5 years. In the base case scenario, HBV disease progression in the form of the 5‐year cumulative incidence of cirrhosis and HCC would be less than 0.5% regardless of TDF/FTC use (Table S4); the cumulative incidence of moderate or severe fibrosis would be 2.0% (*TDF/FTC*) versus 2.8% (*no TDF/FTC*).

**FIGURE 1:**
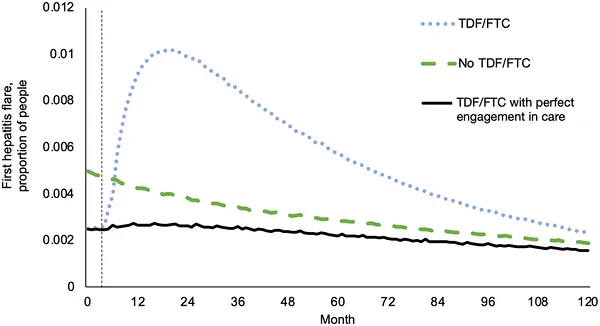
Timing of first hepatitis flare with and without initiation of TDF/FTC as PrEP in women who sell sex living with chronic HBV infection. Hepatitis flares are defined as alanine aminotransferase > two times the upper limit of normal. Simulation starts at time 0 when women initiate TDF/FTC as PrEP (blue dotted line) and no TDF/FTC (green dashed line). A third simulation of women who initiate TDF/FTC and never disengage from care is shown for comparison (solid black line). The vertical dashed line indicates the time (4 months) after which cessation of TDF/FTC could trigger a hepatitis flare in the model.

### 3.3 Sensitivity and Scenario Analyses

In sensitivity analysis, the most influential parameters on 5‐year HIV incidence were the probabilities of disengagement from PrEP care, PrEP adherence and returning to PrEP care. If there were no disengagement of care in 5 years, the cumulative HIV incidence would be 6.0 percentage points lower with *TDF/FTC* compared with *no TDF/FTC* (Figure 2A). Increasing disengagement from care to 18%, lowering PrEP adherence to 30% or lowering the probability of returning to PrEP care to 0% would each diminish the benefits of HIV prevention compared with the base case scenario. However, even with these extreme parameter values, *TDF/FTC* would reduce HIV incidence by at least 1.4 percentage points compared with *no TDF/FTC*.

**FIGURE 2:**
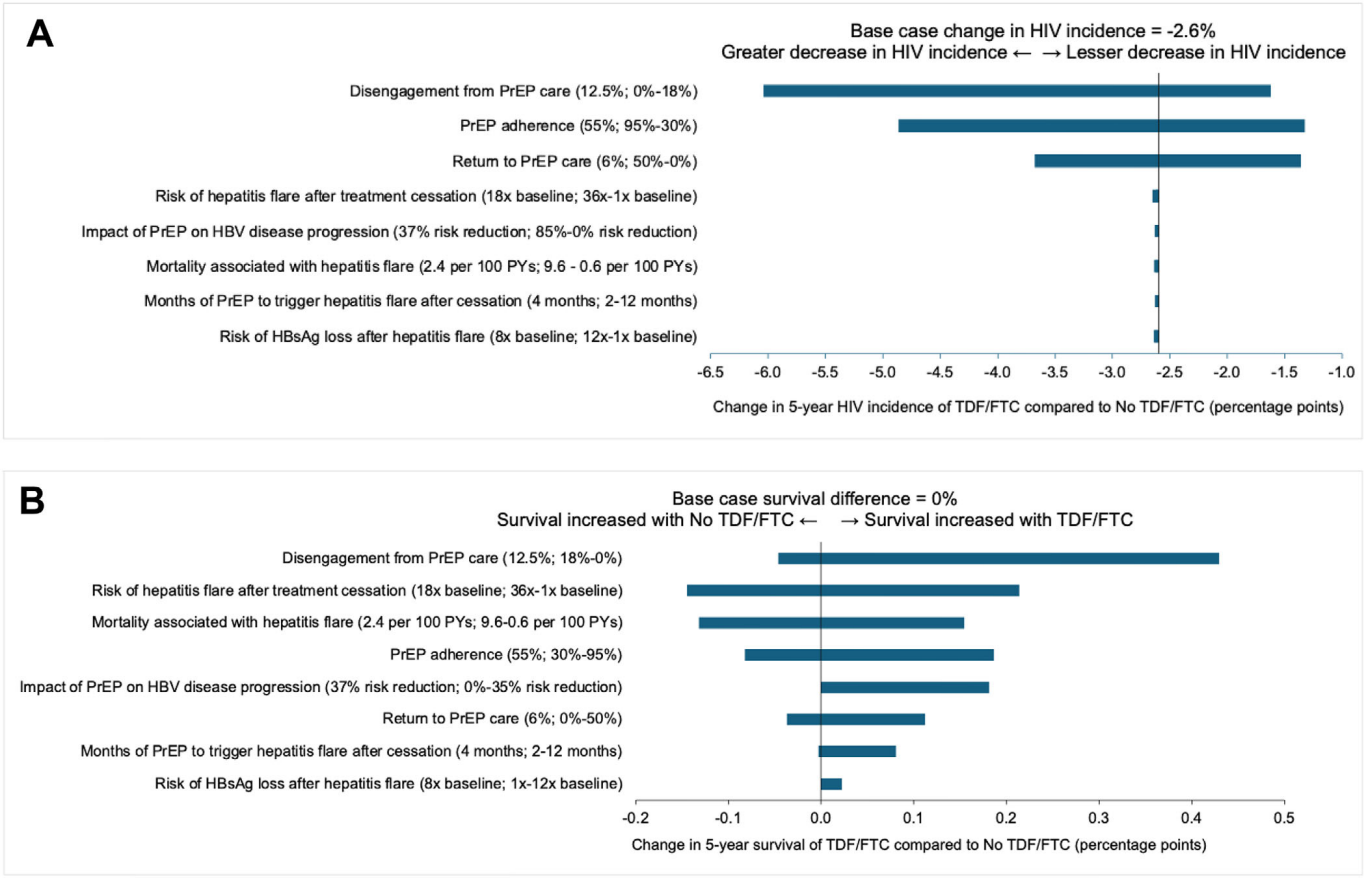
One‐way sensitivity analyses of cumulative HIV incidence (Panel A) and survival (Panel B) at 5 years, comparing *TDF/FTC* versus *no TDF/FTC* as PrEP in women who sell sex with chronic HBV in Côte d'Ivoire. These tornado diagrams demonstrate the effect of a range of parameter values on the outcomes of 5‐year HIV incidence (Panel A) and 5‐year survival (Panel B). On the *x*‐axis of Panel A is shown the change in 5‐year HIV cumulative incidence of *TDF/FTC* compared to *no TDF/FTC* in percentage points. On the *x*‐axis of Panel B is the change in 5‐year survival of *TDF/FTC* compared to *no TDF/FTC* in percentage points. The variables are shown along the vertical axis in decreasing order of influence on the outcome of interest. The base case values are included in the parentheses, followed by the range of values evaluated in sensitivity analyses. The vertical line in both panels represents the base case outcome. Negative values in HIV incidence favour *TDF/FTC* over *no TDF/FTC* (Panel A). Positive values in survival favour *TDF/FTC* over *no TDF/FTC* (Panel B). Parameters and references used in sensitivity analysis are detailed in the Supplementary Material.

The most influential parameter on 5‐year survival was the probability of disengaging from care; most values tested in sensitivity analysis for this parameter resulted in an increase in survival with *TDF/FTC* (Figure 2B). The most extreme value of this parameter to favour *no TDF/FTC* (18% probability of disengaging from care) would only result in a 0.05 percentage point increase in 5‐year survival with *TDF/FTC* compared with *no TDF/FTC*. The other four parameters that demonstrated any improvement in survival with *no TDF/FTC* in sensitivity analysis were the risk of hepatitis flares following PrEP cessation, the mortality associated with a hepatitis flare, PrEP adherence and the probability of returning to PrEP care (Figure 2B).

A two‐way sensitivity analysis demonstrates the potential impact of severe hepatitis flares on 5‐year survival (Figure 3A). If a severe hepatitis flare occurs 10% as frequently as a hepatitis flare defined in the base case scenario and is associated with a 10‐fold higher risk of death, the 5‐year survival with *TDF/FTC* would be similar to the *no TDF/FTC* strategy. Nearly all combinations of parameters examined in this sensitivity analysis would result in the 5‐year survival difference between *TDF/FTC* and *no TDF/FTC* increasing from the base case but remaining within 0.3 percentage points of each other. The cumulative 5‐year incidence of HIV would be between 2.6 and 2.7 percentage points lower with *TDF/FTC*. Simultaneously varying both the risk of hepatitis flare and the risk of HBsAg loss following TDF/FTC cessation would increase the cumulative incidence of HBsAg loss by up to a 6.7 percentage point difference compared with the *no TDF/FTC* strategy (Figure 3B).

**FIGURE 3:**
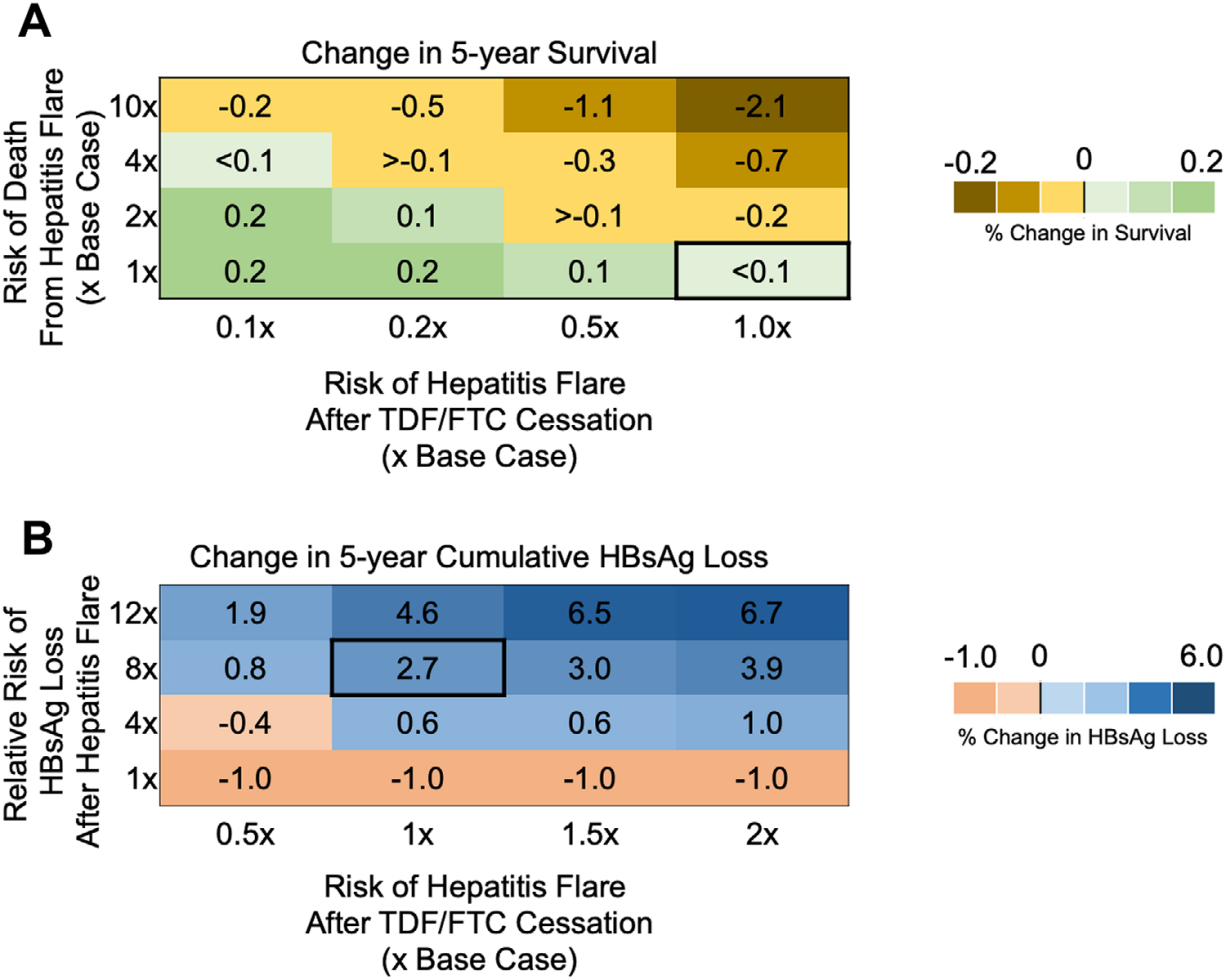
Two‐way sensitivity analyses of (Panel A) hepatitis flare incidence versus hepatitis flare mortality and (Panel B) hepatitis flare incidence and HBsAg loss incidence with 5‐year outcomes of *TDF/FTC* versus *no TDF/FTC* in women who sell sex in Côte d'Ivoire living with chronic HBV. Panel A: Percentage point change in 5‐year survival when varying hepatitis flares’ incidence after TDF/FTC cessation (*x*‐axis) and mortality associated with hepatitis flares (*y*‐axis). Each parameter value on the *x*‐axis and *y*‐axis is a multiple of the value used in the base case. The outlined cell indicates the base case outcome. The positive values (green) favour *TDF/FTC* over *no TDF/FTC*. The negative values (yellow) favour *no TDF/FTC* over *TDF/FTC*. The change in 5‐year survival is similar (within 0.2 percentage points) between the two strategies when there are more severe hepatitis flares (e.g. 10 × the base case value on the *y*‐axis), which occur less frequently (e.g. 0.1 × the base case value on the *x*‐axis) than base case estimates (outlined in black). Panel B: Percentage point change in 5‐year cumulative HBsAg loss while varying the risk of hepatitis flare as a factor of the base case (*x*‐axis) and the risk of HBsAg loss from a hepatitis flare as a factor from the risk prior to hepatitis flare (*y*‐axis). The outlined cell indicates the base case outcome. The bottom row represents a scenario where there is no increased risk of HBsAg loss after a hepatitis flare compared with before a hepatitis flare (i.e. relative risk of HBsAg loss after a hepatitis flare = 1 × the baseline risk). The positive values (blue) show that *TDF/FTC* is preferred compared with *no TDF/FTC*, and the negative values (red) show that *no TDF/FTC* is the preferred strategy. HBsAg, HBV surface antigen loss; TDF/FTC, tenofovir‐emtricitabine.

In a scenario where the mean starting age of the population is 35 years old, 5‐year cumulative HIV incidence would be 8.4% *TDF/FTC* versus 8.7% *no TDF/FTC*, with no difference in 5‐year survival. The 5‐year cumulative incidence of liver disease from HBV would be higher (fibrosis: 4.30% *TDF/FTC* vs. 4.44% *no TDF/FTC*) compared with the base case scenario (fibrosis: 2.00% *TDF/FTC* vs. 2.80% *no TDF/FTC*). Use of TDF/FTC without disengagement from care would improve HBV‐related outcomes by reducing the risk of liver disease (Table S4). Similar results occurred at lower HIV incidence (1.5 per 100 person‐years, decreasing with older age): while overall HIV incidence would be lower, *TDF/FTC* would result in a greater reduction in HIV (cumulative HIV incidence at 5 years: 5.2% *TDF/FTC* vs. 6.6% *no TDF/FTC*) and improved survival (cumulative 10‐year survival: 76.4% *TDF/FTC* vs. 75.9% *no TDF/FTC*). Use of TDF/FTC as PrEP would similarly reduce HIV incidence and improve survival in scenarios with lower background mortality and lower linkage to care after HIV diagnosis (Table S5).

## 4 Discussion

In this study, we found that use of TDF/FTC as PrEP would reduce new HIV acquisition among women who sell sex with chronic HBV in Côte d'Ivoire compared with *no TDF/FTC*. Even with a high rate of disengagement from PrEP care, *TDF/FTC* would avert more than 20% of new HIV acquisitions. Risk of hepatitis flares following TDF/FTC cessation would be mitigated by the substantial benefits of HIV prevention. Survival would favour TDF/FTC as PrEP after 5 years. Continuous TDF/FTC (i.e. no disengagement in care) would yield even greater benefits in terms of HIV prevention and survival without incurring the additional risk of hepatitis flares. This study provides an evidence base for expanding TDF/FTC among priority populations at risk for HIV living with HBV in sub‐Saharan Africa. As people with HBV have been largely excluded from most PrEP clinical trials [11], and as there are few HBV treatment studies underway in sub‐Saharan Africa, these results can guide clinicians and policymakers in planning the expansion of oral PrEP in their health systems [53].

An important consideration for oral PrEP in people with HBV is that increased risk of hepatitis flares and further complications following PrEP cessation could be challenging to manage in resource‐limited settings unless people had access to close monitoring and supportive care [9]. In contrast to high‐income settings, liver transplantation would not be an option in most resource‐limited settings should hepatitis flares progress to acute liver failure. In this analysis, we project that hepatitis flares are likely to be infrequent among women who maintain consistent use of TDF/FTC as PrEP but would increase as more TDF/FTC cessation occurs, surpassing the cumulative incidence for women who never initiate TDF/FTC. However, we conservatively defined a hepatitis flare as an ALT elevated above two times the upper limit of normal, which in most cases would not be clinically apparent, would not require medical treatment and would not result in long‐term liver damage [48]. Results from the two‐way sensitivity analysis provide insight into the potential impact of severe hepatitis flares: less frequent but more deadly hepatitis flares could lower 5‐year survival in *TDF/FTC* in relation to *no TDF/FTC*, but differences would remain small (i.e. less than 0.3 percentage points), and the HIV prevention benefits continue to strongly favour *TDF/FTC* over *no TDF/FTC*.

Safely inducing HBsAg loss in people with HBV is of great interest as a functional cure of chronic HBV because this clinical endpoint is associated with lower rates of liver‐related morbidity and mortality and is a strong indicator for stopping antiviral therapy [54]. Data continue to emerge regarding the potential benefits of targeted treatment cessation strategies in HBV monoinfection [15, 29, 49]. HBsAg loss is likely to be lower after PrEP cessation because TDF/FTC use as PrEP is for a shorter duration of time. It should be emphasized that no published studies have evaluated HBsAg loss after brief periods of TDF/FTC use, such as in PrEP [9, 37]. We found that the *TDF/FTC* strategy would increase cumulative HBsAg loss compared with *no TDF/FTC* in the base case scenario. Even if HBsAg loss did not increase after hepatitis flare, as tested in the sensitivity analysis, *TDF/FTC* would still be preferred as a strategy to improve long‐term survival by reducing the risk of HIV.

Despite the long‐term benefits of TDF/FTC in reducing progression of chronic liver disease in people with chronic HBV, we found that short‐term use of PrEP did not have a major impact on reducing cirrhosis and HCC events in this population. The limited liver‐related benefits are likely due to the brief duration of use and the low baseline risk of hepatic complications within 5 years for this young population of women. More sustained TDF/FTC use would be needed to reduce the incidence of liver‐related complications of chronic HBV. As in most parts of sub‐Saharan Africa, people in Côte d'Ivoire diagnosed with chronic HBV must pay for their own laboratory testing, monitoring and treatment. Therefore, many people are never initiated on therapy for HBV or are lost to follow‐up. Priority populations who have chronic HBV with indications for treatment should be treated with TDF/FTC. Scale‐up of PrEP in people with HBV must also be accompanied by expanded access to TDF/FTC and investment in monitoring for hepatitis flares after TDF/FTC cessation and clinical management.

Closer integration of PrEP and HBV care could allow priority populations to remain engaged in care for HIV prevention and minimize the possible risks of hepatitis flares after TDF/FTC cessation. Monitoring for HBV reactivation and hepatitis flares should be incorporated into PrEP monitoring for priority populations with HBV whenever possible and especially after TDF/FTC cessation [10]. However, as this analysis shows, the absence of consistent monitoring for HBV reactivation should not preclude use of TDF/FTC as PrEP in priority populations because of the substantial benefits of HIV prevention relative to the lower risk of hepatitis flares. There are important distinctions between women who sell sex, as studied in this analysis, and other priority populations. First, we modelled a high incidence rate of HIV acquisition based on source studies from the region. People with a lower risk of HIV acquisition would derive less of a benefit from PrEP, as shown in the scenario analyses. Second, we modelled a high background mortality, consistent with other studies demonstrating the high risk of death related to safety issues for sex workers. Third, we modelled a high rate of disengagement from PrEP care, which would increase the risk of hepatitis flares and reduce the protective effects of preventing HIV acquisition. Sensitivity and scenario analyses demonstrated that more consistent use of PrEP with high adherence and low disengagement from care would maximize both HIV‐ and HBV‐related benefits. Aligning the goals of PrEP programmes with public health initiatives addressing HBV could yield dual benefits, but more efforts would be needed to ensure long‐term engagement in care and medication adherence.

This study has several limitations. Limited data on HBV‐related outcomes from HIV prevention studies result in model input parameters that have some uncertainty. We examined the impact of this uncertainty by varying key input parameters in sensitivity analyses to determine their impact on model outcomes. We did not examine potential benefits of TDF/FTC reducing onward transmission of HIV or HBV to additional sexual partners. Finally, we did not examine the interactions between HIV and HBV coinfection among people who acquire HIV in the simulation model. Despite these limitations, this modelling study provides novel evidence on the benefits of TDF/FTC as PrEP in people with chronic HBV, a population systematically excluded from most PrEP clinical trials and implementation studies.

## 5 Conclusions

These model‐based results support the conclusion that TDF/FTC use would be effective in preventing HIV and improving long‐term survival despite the increased short‐term risk of hepatitis flares following TDF/FTC cessation among women who sell sex living with chronic HBV. The liver‐related harms of using TDF/FTC for brief periods of time in this population would be outweighed by the benefits in HIV prevention, even with conservative assumptions made regarding the severity and frequency of hepatitis flares after TDF/FTC cessation. The benefits of using TDF/FTC to prevent liver‐related complications of HBV, such as cirrhosis and HCC, would be less apparent with the brief duration of use and limited adherence observed in most PrEP implementation studies. Given the lack of data on PrEP among priority populations living with HBV, this study provides evidence supporting the scale‐up of oral PrEP programmes for people living with HBV and integrating efforts at HIV prevention and HBV monitoring in HBV‐endemic settings.

## Author Contributions

**Amir M. Mohareb**: Conceptualization, investigation, Writing – original draft, funding acquisition, validation, visualization, Writing – review and editing, formal analysis, software. **Arthur Y. Kim**: Investigation, funding acquisition, writing – review and editing, supervision, formal analysis. **Menan Gérard Kouamé**: Investigation, writing – review and editing, formal analysis. **Marcellin Nouaman**: Investigation, writing – review and editing. **Patrick A. Coffie**: Investigation, writing – review and editing. **Maya Hajny Fernandez**: Investigation, writing – review and editing, validation, methodology, software. **Virginia Talbot**: Investigation, methodology, software, validation, formal analysis, writing – review and editing. **Ruitian Hu**: Investigation, validation, software, formal analysis, writing – review and editing, visualization, project administration. **Anne M. Neilan**: Investigation, writing – review and editing, formal analysis. **Anders Boyd**: Investigation, methodology, writing – review and editing, formal analysis, funding acquisition. **Kenneth A. Freedberg**: Investigation, validation, writing – review and editing, formal analysis, software, methodology, supervision, resources, project administration. **Joseph Larmarange**: Investigation, writing – review and editing, funding acquisition, methodology, formal analysis, software, data curation, supervision. **Emily P. Hyle**: Investigation, funding acquisition, validation, methodology, writing – review and editing, visualization, software, formal analysis, project administration, supervision, resources.

## Funding

This work is partially supported by the National Institute for Allergy and Infectious Diseases (NIAID) at the National Institutes of Health (NIH) (grant number K01AI166126 to AMM; grant number R37AI058736 to KAF), the Harvard University Center for AIDS Research (grant number P30AI060354 to AMM), the Eunice Kennedy Shriver National Institute of Child Health and Human Development at the NIH (grant number R01HD111355 to AMN), the Massachusetts General Hospital (MGH) Department of Medicine Transformative Scholars Award (grant to AMN), and the Jerome and Celia Reich Endowed Scholar in HIV/AIDS Research at MGH (grant to EPH). The PRINCESSE project was funded by the ANRS|MIE (the French National Research Agency for AIDS, Viral Hepatitis and Emerging Infectious Diseases, http://www.anrs.fr, grant 12381), which also sponsored the study. The funders had no role in the design or authorship of this publication.

## Conflicts of Interest

AB reports receiving speaker fees from Gilead Sciences, Inc.

## Disclaimer

The article contents are solely the responsibility of the authors and do not necessarily represent the official views of the funders.

## Supporting information




**Supporting file 1**: jia270217‐sup‐0001‐SuppMat.docx

## Data Availability

Data that support the findings of this study are outlined in the Supplementary Material of this article. Additional data that support the findings of this study, including detailed model specifications, are available from the corresponding author upon reasonable request.
