# Supplementary material for: Impact of HIV Pre‐Exposure Prophylaxis on HIV Incidence and Hepatitis B Outcomes Among Sex Workers Living With Hepatitis B: A Modelling Analysis in Côte D'Ivoire
Source: J Int AIDS Soc. 2026 Oct 26;29(10):e70217. doi: 10.1002/jia2.70217 (PMC13697507; doi:10.1002/jia2.70217)
Supplement: Supplementary file 1 [file JIA2-29-e70217-s001.docx]

**Supplement to**:

Impact of HIV Pre-Exposure Prophylaxis (PrEP) on HIV incidence and hepatitis B outcomes among sex workers living with hepatitis B: a modeling analysis in Côte d’Ivoire

**Authors**

Amir M. Mohareb, Arthur Y. Kim, Menan Gérard Kouamé, Marcellin Nouaman, Patrick Coffie, Maya Hajny Fernandez, Virginia Talbot, Ruitian Hu, Anne Neilan, Anders Boyd, Kenneth A. Freedberg, Joseph Larmarange, Emily P. Hyle

**Supplement Table of Contents:**

Supplement Table 1: Studies relevant to the increased risk of death in women who sell sex compared with the general population.

Supplement Table 2: Studies relevant to evaluating PrEP loss-to-follow-up in women who sell sex in sub-Saharan Africa.

Supplemental Table 3: Parameter values used in sensitivity analyses for simulation modeling study of TDF/FTC versus No TDF/FTC as HIV pre-exposure prophylaxis in women who sell sex with chronic HBV in Côte d’Ivoire

Supplement Table 4: Simulated liver-related outcomes with TDF/FTC versus no TDF/FTC.

Supplement Figure 1: Health states in HIV-HBV coinfection simulation model

Supplement Figure 2: Disengagement from PrEP care in women who sell sex in sub-Saharan Africa

Supplement Figure 3: Simulated 10-year survival of sex workers with chronic HBV with TDF/FTC as PrEP versus no TDF/FTC.

**Supplement Table 1**: Studies relevant to the increased risk of death in women who sell sex compared with the general population.

| **First Author (Year)** | **Setting** | **Study Design** | **Key Findings** |
| --- | --- | --- | --- |
| Potterat (2004)^1^ | United States | Longitudinal observational cohort study | Standardized mortality ratio 5.9 (95% CI: 3.2 – 9.0) compared with general population.  Standardized mortality ratio from homicide 17.7 (95% CI: 6.2 – 29.3) compared with general population. Other causes of death included suicide, accident, HIV, and substance use. |
| Huet (2011)^2^ | Burkina Faso | Prospective observational cohort study | Among sex workers with HIV, 81.8% (95% CI: 59.7 – 94.8%) had undetectable viral load compared with 100% (95% CI: 83.9% - 100%) of non-sex workers after 36 months of follow-up.  Mortality rate among sex workers with HIV was 1.7 per 100 person-years. |
| Lujintanon (2024)^3^ | South Africa | Secondary analysis of clinical trial | Physical and sexual violence was associated with being lost to follow-up. Risk of death after 18 months was 3.7% (95% CI: 1.8% - 5.9%). |
| Willis (2022)^4^ and Macias-Konstantopoulos (2024)^5^ | Multi-country | Community knowledge approach | Causes of death of 2,112 sex workers ascertained through community interviews included maternal mortality (52.1%), suicide (13.6%), homicide (12.5%), unknown (11.6%), HIV (7.9%), and accidents (3.2%). Most died between ages 20- 29 years-old. |

**Supplement Table 2**: Studies relevant to evaluating PrEP retention in care in women who sell sex in sub-Saharan Africa.

| **First Author (Year)** | **Setting** | **Retention in care** |
| --- | --- | --- |
| Sarr (2020)^6^ | Senegal | 73.4% at 12 months |
| Mboup (2018)^7^ | Benin | 11.7% at 24 months |
| Eakle (2017)^8^ | South Africa | 9.6% at 24 months |
| PRINCESSE (2024)^9^ | Côte d’Ivoire | 14% at 39 months |

**Supplemental Table 3**: Parameter values used in sensitivity analyses for simulation modeling study of *TDF/FTC* versus *No TDF/FTC* as HIV pre-exposure prophylaxis in women who sell sex with chronic HBV in Côte d’Ivoire

| **Parameter** | **Range in sensitivity analysis** | **Reference** |
| --- | --- | --- |
| PrEP Adherence | 30 - 95% | Murchu (2022)^10^ |
| Probability of disengaging from PrEP care | 0.0 – 0.18 | See Supplemental Table 2 and Supplemental Figure 2 |
| Probability of returning to PrEP care | 0.0 – 0.50 | See Supplemental Figure 2 |
| Risk of hepatitis flare following TDF/FTC cessation | No additional risk – 36 x the baseline risk in the month following cessation | Dongelmans (2025)^11^ |
| Mortality associated with hepatitis flare | 0.25 x base case – 4 x base case | Ghany (2020)^12^ |
| Relative risk of HBsAg loss following hepatitis flare | 1.0 – 12.0 x the baseline risk | Hall (2022)^13^ |
| Impact of TDF/FTC on HBV disease progression | 0- 85% risk reduction | Model assumption |
| Duration of PrEP to trigger a hepatitis flare after cessation | 2- 12 months | Model assumption |

**Supplement Table 4**: Five-year liver-related outcomes in simulation modeling study of *TDF/FTC* versus *No TDF/FTC* as HIV pre-exposure prophylaxis in women who sell sex with chronic HBV in Côte d’Ivoire

| **Strategy** | **Fibrosis**  **(%)** | **Cirrhosis**  **(%)** | **HCC**  **(%)** | | **ESLD**  **(%)** |
| --- | --- | --- | --- | --- | --- |
|  | 5-year | 5-year | 5-year | 5-year | |
| **Base Case** |  |  |  |  | |
| No TDF/FTC | 2.80% | 0.28% | 0.04% | 0.09% | |
| TDF/FTC | 2.00% | 0.23% | 0.04% | 0.08% | |
| **Sensitivity Analyses** | | | | | |
| TDF/FTC with no disengagement in care | 1.38% | 0.12% | 0.03% | 0.06% | |
| Mean starting age 35y: No TDF/FTC | 4.44% | 0.57% | 0.12% | 0.16% | |
| Mean starting age 35y: TDF/FTC | 4.30% | 0.54% | 0.12% | 0.15% | |

Abbreviations:

HCC: hepatocellular carcinoma; ESLD: end-stage liver disease; TDF/FTC: tenofovir disoproxil fumarate/ emtricitabine

**Supplement Table 5**: Scenario analyses of five-year outcomes of *TDF/FTC* versus *No TDF/FTC* as HIV pre-exposure prophylaxis in women who sell sex with chronic HBV in Côte d’Ivoire

| **Strategy** | | **HIV Incidence**  **(%)** | | | | **HBsAg Loss**  **(%)** | | | | **Survival**  **(%)** | | | |
| --- | --- | --- | --- | --- | --- | --- | --- | --- | --- | --- | --- | --- | --- |
|  | | 2-year | | 5-year | | 2-year | | 5-year | | 2-year | | 5-year | 10-year |
| **Base Case Scenario** | | | | | | | | | | | | | |
| No TDF/FTC | 5.7 | | 12.7 | | 2.3 | | 5.7 | | 96.2 | | 89.4 | | 72.6 |
| TDF/FTC | 4.1 | | 10.1 | | 2.9 | | 8.4 | | 96.2 | | 89.4 | | 73.8 |
| **Scenario: Lower HIV incidence rate (1.5 per 100 person-years at age 25 years old)** | | | | | | | | | | | | | |
| No TDF/FTC | | 2.9 | | 6.6 | | 2.3 | | 5.7 | | 96.3 | | 89.2 | 75.9 |
| TDF/FTC | | 2.1 | | 5.2 | | 2.9 | | 8.4 | | 96.3 | | 89.9 | 76.4 |
| **Scenario: Lower background mortality (no RMR applied)** | | | | | | | | | | | | | |
| No TDF/FTC | | 5.8 | | 13.2 | | 2.4 | | 5.9 | | 99.4 | | 97.7 | 88.9 |
| TDF/FTC | | 4.2 | | 10.5 | | 3.0 | | 8.7 | | 99.4 | | 97.8 | 90.3 |
| **Scenario: Lower linkage to care (75%) after HIV diagnosis** | | | | | | | | | | | | | |
| No TDF/FTC | | 5.7 | | 12.7 | | 2.3 | | 5.7 | | 96.3 | | 89.6 | 75.4 |
| TDF/FTC | | 4.1 | | 10.1 | | 2.9 | | 8.3 | | 96.3 | | 89.6 | 76.0 |

Abbreviations:

HBsAg: hepatitis B virus surface antigen; RMR: relative mortality ratio; TDF/FTC: tenofovir disoproxil fumarate/ emtricitabine

Uninfected

Infected

Acute Event

Death

Uninfected

Infected

Acute Event

Death

**Supplement Figure 1**: Health states in HIV-HBV coinfection simulation model.

HIV and hepatitis B health states advance in parallel each month. All simulated persons start with hepatitis B (left) and have a risk of also acquiring HIV coinfection (right). Use of TDF/FTC as PrEP decreases the risk of HIV acquisition and reduces the probability of advancing through hepatitis B health states (i.e., lower probability of developing fibrosis, cirrhosis, HCC, end-stage liver disease, or a hepatitis flare). Use of TDF/FTC as ART for those who acquired HIV reduces the risk of acute events in HIV, such as opportunistic infections. Hepatitis Flare is modeled as an acute event in the simulation. Death is the terminal state (not shown), and all health states include a risk of death with an increased risk among some health states.

Abbreviations: HCC: hepatocellular carcinoma; PrEP: pre-exposure prophylaxis; ART: antiretroviral therapy; TDF/FTC: tenofovir disoproxil fumarate / emtricitabine

**Supplement Figure 2**: Disengagement from PrEP care among women who sell sex

Data from studies at different time points are shown in black (see Supplemental Table 2 for references). Simulated estimates of disengagement from care are shown in colored lines. Dotted lines have a simulated change in the probability of disengaging from care at 6 months. Solid lines have a simulated change in the probability of disengaging from care at 12 months. Gray solid line (4% per month up to 12 months then 12.5% per month) represents the base case scenario used in the modeling analysis. Other lines demonstrate the values used in sensitivity analyses.

**Supplement Figure 3**: Simulated 10-year survival of women who sell sex with chronic HBV with *TDF/FTC* as PrEP versus *No TDF/FTC*.

TDF/FTC as PrEP with 100% retention (i.e., no disengagement from care) is shown in solid black line for comparison. The markedly increased risk of death among women who sell sex compared with the general population was modeled as a relative mortality ratio of 8.0 to the background age-stratified mortality risk for women in Côte d’Ivoire (see Supplement Table 1).

Abbreviation: TDF/FTC: tenofovir disoproxil fumarate/emtricitabine

**References to Supplement**

1. Potterat JJ, et al. Mortality in a long-term open cohort of prostitute women. *Am J Epidemiol*. 2004;159(8):778-785.

2. Huet C, et al. Long-term virological, immunological and mortality outcomes in a cohort of HIV-infected female sex workers treated with highly active antiretroviral therapy in Africa. *BMC Public Health*. 2011;11:700.

3. Lujintanon S, et al. Estimating the mortality risk correcting for high loss to follow-up among female sex workers with HIV in Durban, South Africa, 2018-2021. *Ann Epidemiol*. 2024;92:8-16.

4. Willis B, et al. Causes of mortality among female sex workers: Results of a multi-country study. *EClinicalMedicine*. 2022;52:101658.

5. Macias-Konstantopoulos WL, et al. Causes of preventable death among children of female sex worker mothers in low- and middle-income countries: A community knowledge approach investigation. *J Glob Health*. 2024;14:04052.

6. Sarr M, et al. Uptake, retention, and outcomes in a demonstration project of pre-exposure prophylaxis among female sex workers in public health centers in Senegal. *Int J STD AIDS* 2020;31:1063–72.

7. Mboup A, et al. Early antiretroviral therapy and daily pre-exposure prophylaxis for HIV prevention among female sex workers in Cotonou, Benin: a prospective observational demonstration study. *J Int AIDS Soc* 2018;21:e25208.

8. Eakle R, et al. HIV pre-exposure prophylaxis and early antiretroviral treatment among female sex workers in South Africa: Results from a prospective observational demonstration project. *PLoS Med* 2017;14:e1002444.

9. PRINCESSE follow-up. Available from: <https://hal.science/ANRS_12381_PRINCESSE/hal-04609403v1>. Accessed 6 October 2025.

10. O Murchu E, et al. Oral pre-exposure prophylaxis (PrEP) to prevent HIV: a systematic review and meta-analysis of clinical effectiveness, safety, adherence and risk compensation in all populations. *BMJ Open*. 2022;12(5):e048478.

11. Dongelmanns EJ, Predictors of hepatic flares after nucleos(t)ide analogue cessation - Results of a global cohort study (RETRACT-B study). *J Hepatol*. 2025;82(3):446-455.

12. Ghany M, Serum alanine aminotransferase flares in chronic hepatitis B infection: the good and the bad. *Lancet Gastroenterol Hepatol* 2020;5(4):406-417.

13. Hall SAL, et al. Discontinuation of nucleot(s)ide analogue therapy in HBeAg-negative chronic hepatitis B: a meta-analysis. *Gut*. 2022;71(8):1629-1641.
